# A function-based framework for AI-amplified, data-driven healthcare simulation research

**DOI:** 10.1186/s41077-026-00426-x

**Published:** 2026-02-28

**Authors:** Carla Sa-Couto

**Affiliations:** RISE-Health, Faculty of Medicine, University of Porto, Al. Prof. Hernâni Monteiro, Porto, 4200–319 Portugal

**Keywords:** Simulation-based research, Artificial intelligence, Framework, Data-Driven Research, Healthcare Simulation

## Abstract

Healthcare simulation research increasingly generates rich, multimodal datasets, yet substantial portion of these data remain underutilized due to analytic, technical, and resource constraints. As artificial intelligence becomes more accessible, there is a risk that adoption in simulation research is driven by novelty rather than by clearly articulated analytic needs. This article proposes a function-based framework that conceptualizes artificial intelligence as a *research amplifier* for data-driven simulation-based research. Rather than organizing approaches by algorithm family or tool type, the framework groups AI use according to its function in the research workflow across four domains: (1) Data Processing and Integration: transforming raw traces into synchronized, research-ready datasets; (2) Comparative and Predictive Analytics: benchmarking performance and modeling learning trajectories or outcomes; (3) Behavioral and Interaction Analysis: decoding individual and team behaviors from observational traces; and (4) Automation and Acceleration: streamlining research activities such as transcription, coding support, and structured summarization through automated or semi-automated workflows. Five practical steps illustrate how the framework can be embedded in research planning: clarifying the analytic task, mapping it to functional domains, specifying data representations and integration needs, designing the human–AI division of labor, and planning evaluation and reporting. Ethical, trust, and accountability considerations are discussed across functional domains, emphasizing that distinct risks and safeguards emerge across different AI-enabled research activities. By centering function and purpose, this framework aims to help simulation researchers make explicit, intentional decisions about when and how AI meaningfully extends analytic capacity.

**Supplementary Information:**

The online version contains supplementary material available at 10.1186/s41077-026-00426-x.

## Background

Healthcare simulation research has evolved dramatically in its capacity to generate comprehensive datasets, yet paradoxically faces an expanding gap between data collection and analytical utilization. Contemporary simulation environments routinely produce large volumes of data spanning from structured measures (e.g., checklists, rating scales, simulator-derived metrics, event logs) to unstructured or high-frequency traces (e.g., audio, video, physiological monitoring, eye tracking, positioning/movement) [1–4]. Compared with other educational settings, healthcare simulations often involve high-acuity, time-pressured scenarios in which interprofessional teams must coordinate communication, spatial positioning, and technical actions [2, 5]. Despite this wealth of information, only a fraction of available data is typically processed, analyzed, and used for educational or research purposes. Several studies [5–7] in multimodal and simulation-adjacent learning analytics highlight this underutilization, reporting that substantial portions of simulation data remain unexamined due to analytic and resource limitations. Similar patterns have been documented in healthcare more broadly: real-world data stored in clinical systems and shared clinical research datasets remain substantially underused for secondary analysis and knowledge generation, with many available datasets never requested or reused [8–10].

Several obstacles contribute to this analytic bottleneck. Simulation datasets often require substantial data processing before they become analyzable. Simulation data are inherently heterogeneous, and integrating diverse data sources into a coherent, structured dataset poses methodological and technical challenges [4, 11]. Data quality concerns, such as synchronization errors across modalities, missing or inconsistent metadata, and sensor malfunctions, further complicate data integration and may reduce confidence in findings. Addressing these problems typically requires intensive data monitoring and extensive manual preprocessing [12]. Additionally, the volume and complexity of some data streams (such as continuous physiological monitoring or longitudinal video recordings) often exceed what research teams can feasibly process, without specialized expertise in data science, signal processing, or software engineering [4, 13]. At a broader level, this contributes to many studies remaining narrowly scoped, focusing exclusively on the data required to answer a predefined hypothesis, leaving potentially valuable data unused and limiting opportunities for secondary analysis, longitudinal integration, or discovery-driven research [4].

This tension, between abundant data and limited analytic capacity, creates a need for approaches that can extend human cognitive and technical capabilities. Artificial intelligence (AI) has recently been described as a “threshold” concept in healthcare education and simulation, with the potential to reshape how data are processed, interpreted, and used once it becomes embedded in routine research practice [14]. At the same time, as the role of AI in healthcare simulation research continues to expand, there is a growing need to ensure that its adoption is intentional, justified, and aligned with research objectives, rather than driven by novelty or convenience [14–16].

To support purposeful application, this paper proposes a framework addressing specific analytical challenges encountered in data-driven simulation-based research. Building on the model proposed by Cheng and McGregor [15], which conceptualizes AI applications in healthcare simulation, this paper situates itself within the domain of AI-driven data collection and analysis in simulation-based research. Specifically, it proposes a function-based framework for the conceptual use of AI as a research amplifier, aiming to guide simulation researchers in making purposeful choices about when and how to deploy AI within their studies. Rather than a finalized or prescriptive method, this paper should be read as an essay that invites critique and reflection on the proposed conceptual model, with the intention of enabling its further refinement and empirical testing.

## AI as a tree metaphor

As AI tools become increasingly widespread and accessible, technical terminology has entered the common lexicon, yet there remains a recognized lack of literacy within the simulation community regarding how AI systems function [14]. This section provides foundational clarity on AI-related terminology and key concepts as a primer for the subsequent sections. It is not intended to serve as a comprehensive overview of AI, but rather to offer essential background to support an informed reading of the proposed framework.

AI is the scientific discipline focused on creating systems capable of tasks that typically require human intelligence [17, 18]. At the core of most contemporary AI systems is machine learning (ML), a family of methods in which algorithms learn patterns from data to improve performance on a given task (e.g., classification, prediction, clustering) rather than being explicitly programmed with fixed rules [18]. A wide range of modeling techniques exists within ML, and their selection should be guided by the research question and the characteristics of the available data [18]. The recent advent of deep learning (DL) - a class of ML methods based on multi-layered neural networks - has significantly expanded AI’s capabilities, giving rise to powerful AI systems, including large language models (LLMs) and generative AI tools that are now widely accessible.

Among AI core subfields, natural language processing (NLP) and computer vision (CV) are particularly relevant to simulation research. NLP focuses on enabling computers to understand, interpret, generate, and interact using human language [19]. It combines methods from linguistics and computer science to process and analyze text or speech data [19], supporting tasks such as transcription, classification, information extraction, and summarization. In simulation research, NLP can be used to automatically transcribe verbal interactions (during scenarios or debriefings), extract communication patterns, classify utterances (e.g., closed-loop communication, questions, clarifications), or summarize free-text survey responses and reflective narratives for further analysis [20–22]. CV enables machines to perceive and interpret visual information. It develops algorithms that allow computers to analyze images and videos to detect and recognize objects, track movement, identify patterns, and make decisions based on visual input [23]. In simulation, CV techniques can support automated measurement of posture or hand movements, learner–patient proximity, gaze direction or body orientation, and task-critical actions during procedures or team-based scenarios [24, 25]. Beyond NLP and CV, a broader ecosystem of AI application domains is relevant for diverse simulation data types. For simplicity, these will be considered as o*ther domains*, and include time-series and signal analysis (for physiological waveforms and continuous monitor data), or process or event-log analytics (e.g., sequential models for high-frequency simulator logs) [26].

**Fig. 1:**
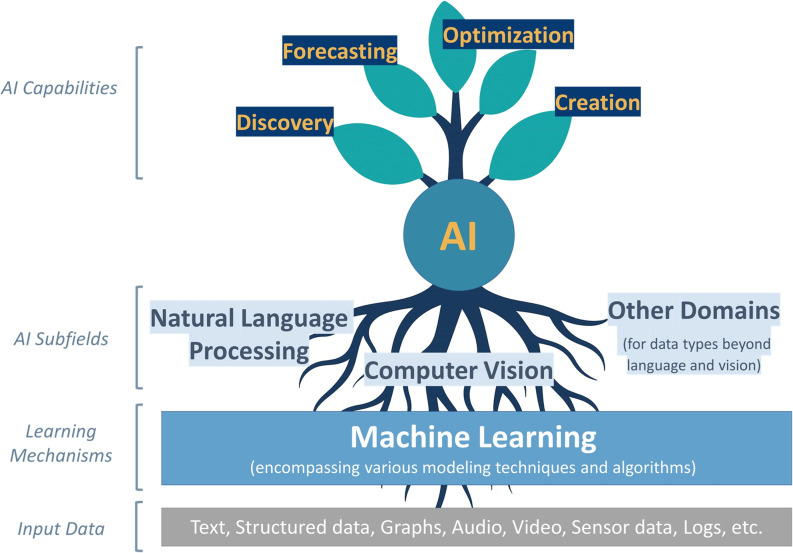
Conceptual overview of artificial intelligence (AI) using a tree metaphor

Across these subfields, AI systems can learn from a multitude of data sources to support a range of research purposes, including *discovering* patterns and relationships in complex datasets, *forecasting* future performance or outcomes, *optimizing* processes or designs, and *creating* new outputs [27, 28]. Conceptually, these relationships can be visualized using a tree metaphor (Fig. 1). Diverse data sources form the soil and nutrients; ML models form the roots and trunk that transform raw data into structured representations; the branches represent functional uses of these representations (e.g., preparation, interpretation, modeling); and the leaves depict higher-level capabilities that emerge from these processes.

## Function-based framework for data-driven AI-amplified simulation-based research

**Fig. 2:**
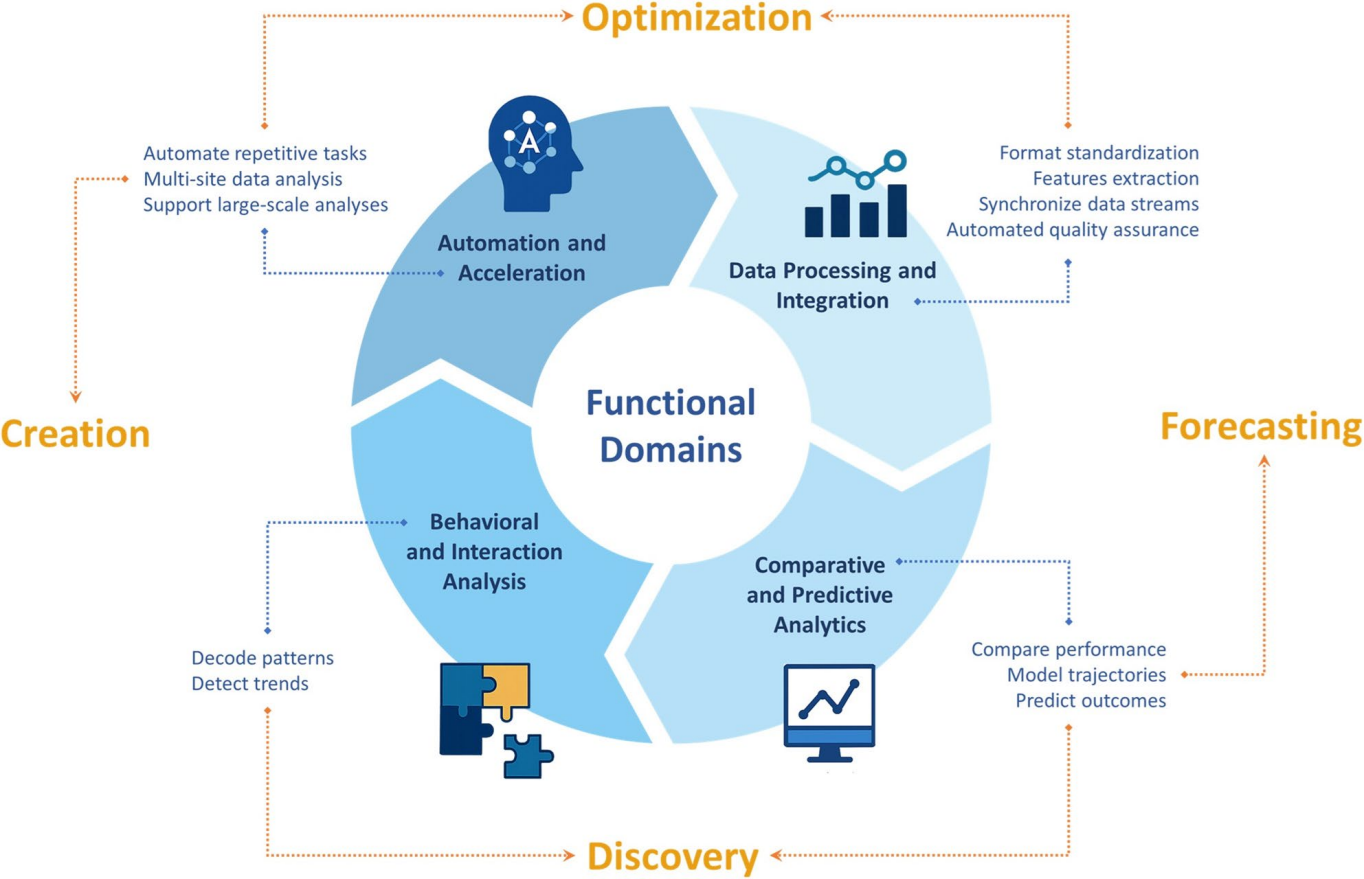
Function-based framework linking AI capabilities with functional domains in data-driven simulation-based research. The inner ring depicts four functional domains in which AI can support simulation research, with example applications indicated in the callouts. The outer labels represent generic, high-level AI capabilities that can be expressed through one or more functional domains

The development of this framework was informed by a targeted, exploratory examination of contemporary literature across simulation-based education, multimodal learning analytics, and AI-in-education research. Relevant papers were identified through focused searches and citation tracking. Across these intersecting fields, four recurring “clusters” of AI-enabled work were consistently identified: (1) transforming raw signals into analyzable representations and integrating multiple data streams [4, 21, 26, 29–31]; (2) comparing performance and predicting outcomes from simulation metrics [29, 32–34]; (3) analyzing behavior and interaction patterns from observational traces [7, 22, 24]; and (4) automating research activities [20, 35–39]. These clusters motivated four corresponding functional domains: Data Processing and Integration, Comparative and Predictive Analytics, Behavioral and Interaction Analysis, and Automation and Acceleration, which organize AI use according to *what it does in the research workflow*, rather than by algorithm family or tool type (Fig. 2). This design choice is intended to keep the framework stable despite fast-moving changes in models, platforms, and nomenclature. Overall, the framework encourages researchers to begin by clarifying the analytic problem and the functional domain, and only then determine whether (and how) AI methods meaningfully contribute to that purpose.

Importantly, not all studies will involve all four domains. For research projects that draw on multiple data streams (e.g., video, audio, logs, physiological signals), Data Processing and Integration often acts as an enabling layer by preparing synchronized, standardized and quality-assured datasets on which the other analytical domains operate. In contrast, projects that work with a single data modality may rely primarily on Comparative and Predictive Analytics, Behavioral and Interaction Analysis, or Automation and Acceleration without a dedicated integration step. Automation and Acceleration can also function as a stand-alone domain (when the central objective is to automate repetitive analytic tasks) or as a cross-cutting enabler for the other domains.

At a more abstract level, the four functional domains can be understood as the mechanisms through which the broader, high-level AI capabilities emerge (Fig. 2). *Discovery* can be achieved through Behavioral and Interaction Analysis, which uses AI to decode patterns and detect trends in communication, workflow, and task execution, and through Comparative and Predictive Analytics, when these patterns and structures are compared across individuals, teams, or conditions. *Forecasting* arises primarily from Comparative and Predictive Analytics, where AI models estimate future performance, learning trajectories, or outcome probabilities from current simulation data. *Optimization* is realized through both Data Processing and Integration and Automation and Acceleration: in data processing and integration, AI is used to optimize the conversion of raw signals into analyzable representations, synchronization and integration of multimodal datasets, and quality assurance of data, whereas in automation, AI helps to design efficient analytic workflows, prioritize which sessions or data streams require human review, and determine minimal yet sufficient measurement strategies. *Creation* mainly follows from Automation and Acceleration, as generative and assistive AI tools are used to produce debriefing summaries, coding suggestions, draft scenarios, or synthetic datasets that extend and accelerate conventional analytic practices.

## Functional domains

### Data processing and integration

This foundational function addresses the challenge of converting raw simulation traces into coherent, analyzable datasets. Simulation environments can generate diverse data sources, including simulator performance metrics, participants’ positioning and movement, verbal communication, eye-tracking, and physiological or biometric signals [4, 30]. In many studies, this heterogeneous source of data is collected simultaneously (often at the team level), increasing the complexity of data alignment, synchronization, standardization, and interpretation [4, 29, 40]. Work in multimodal learning analytics commonly frames this as a pipeline in which raw signals are converted to features and, where relevant, synchronized and fused into a research-ready dataset [30, 31, 40]. Typical processing and integration steps include format transformation (e.g., convert audio waveforms into automatic speech recognition transcriptions, video into pose or body-orientation estimates, and unstructured logs into structured event sequences), feature extraction (e.g., defining phases, tasks, and analytic windows), temporal alignment and synchronization (e.g., synchronize video timestamps with physiological data or positioning data), and automated quality assurance (e.g., detecting sensor failures, missing data segments, or outliers). While many processing operations can be implemented with rule-based or signal-processing methods, contemporary pipelines increasingly rely on ML, particularly DL models such as convolutional neural networks for pose and gesture estimation and automatic speech recognition for speech, to denoise signals, extract higher-level features, and generate structured representations (e.g., who is speaking when; action or gesture traces). Adopting AI for data processing and integration can be technically challenging, as complex pipelines will require input from software engineering or data science experts [4]. Some published multimodal systems are custom in-house developments, limiting their generalizability [7, 26]. Conversely, some commercial platforms (e.g., simulators, audio-visual systems, eye-tracking devices) already incorporate synchronization and embedded ML-based feature extraction (e.g., automatic event logs or gaze metrics), lowering technical barriers. Because complex metrics and pipelines can strain limited resources, simple, feasible methods should be prioritized when they adequately address the research question(s) [4]. Research teams should make deliberate decisions about which data streams to capture, process, and integrate, balancing the analytic value of added complexity against available expertise, time, and infrastructure (see also *From concept to application: Using the framework as a planning tool* section).

Several simulation-based projects exemplify how AI can be used in a supporting data processing and integration role to generate datasets ready for the subsequent analytic stages. For instance, Zhao et al. [7] developed METS (Multimodal Embodied Teamwork Signature), which combines indoor positioning, temporal markers of dialogue, and team-level annotations to generate signatures of embodied teamwork during healthcare simulations. Using a headset microphone and a positioning tag per participant, a custom-built data-capture platform controlled all devices and automatically synchronized multi-channel audio and positioning streams in real time. Voice-activity detection and positioning analytics were then applied to the synchronized data to align who was speaking, where they were located, and how team configurations evolved over time, yielding “teamwork signatures” that educators could visualize and compare across scenarios.

Building on this, Echeverria et al. [26] introduced TeamVision, an AI-powered multimodal learning analytics system designed to support debriefing in team-based healthcare simulations. TeamVision uses a shared capture architecture in which participants wear wireless lapel microphones and positioning sensors, while room cameras record the scenario. All audio and spatial streams are captured and synchronized by a common software layer that also records phase markers entered by an observer. From these synchronized streams, the system derives higher-level features such as voice presence over time, automatically generated speech transcripts, body rotation, and individual trajectories. These features are fused into integrated datasets that can feed a variety of analytics and visualizations during debriefing, while also being reusable for longitudinal research.

A simpler but conceptually similar example is provided by Brutschi et al. [21], who developed an audio-based algorithmic toolkit for analyzing simulation debriefings. In their approach, a single room microphone is used to record debriefings, and a speaker-diarization pipeline, based on open-source ML algorithms, is applied to the raw audio to denoise the signal and segment it into speaker-labeled intervals. The resulting output is a structured, time-indexed sequence of “*who spoke when*,” after which interaction analysis can be conducted using straightforward quantitative measures (e.g., total speaking time, turn-taking counts, response rates) and presented as sociograms.

### Comparative and predictive analytics

This functional domain uses data to assess current performance and to model or predict future outcomes, at the level of individuals, teams or cohorts. AI models are used to compare performance across expertise levels, training conditions or sites, and to forecast outcomes such as proficiency, error risk or learner progression [29, 32–34]. Of note is that, in many cases, the data used in this function have previously gone through Data Processing and Integration. Common analytic approaches range from interpretable methods (e.g., logistic regression, decision trees) to higher-capacity ML models (e.g., support vector machines, k-nearest neighbors), applied to structured metrics, such as task times, error counts, motion paths, force metrics, among others. By exploiting large datasets of repeated simulation attempts, these models can benchmark learner trajectories, stratify risk, and generate targeted feedback. Such predictive capabilities extend beyond descriptive analysis, allowing proactive identification of training needs and early detection of performance trends.

Several neurosurgical simulation studies illustrate these possibilities. Mirchi et al. [33] applied ML algorithms to metrics from a virtual reality (VR) tumor resection simulator to distinguish novice from skilled participants. A feature selection process identified four high-impact metrics: two related to patient safety (e.g., bleeding rate, maximum applied force) and two related to instrument motion. A support vector machine classifier trained on these features achieved approximately 92% overall accuracy, with 100% sensitivity for expert performers and 82% specificity for novices. This study demonstrated that behavioral signatures extracted from simulation data can reliably predict expertise level.

Winkler-Schwartz et al. [34] further demonstrated that ML can be used to classify more granular levels of surgical expertise. Using data from a similar VR brain-tumor resection task, their algorithms analyzed motion, force, resection efficiency, and error metrics from 50 participants ranging from medical students to attending neurosurgeons. A k-nearest neighbors algorithm (a supervised ML) achieved 90% accuracy in classifying participants into four distinct proficiency levels (attending, fellow/senior resident, junior resident, and medical student), illustrating how AI-driven comparative analytics can move beyond binary classification to model the continuum of clinical proficiency, rather than simple novice–expert dichotomies.

More recent work by Loukas and Prevezanou [32] extended this approach to track training progression across three laparoscopic VR tasks in a program followed by 23 medical students. Using simulator-generated performance metrics (task time, instrument pathlength, number of movements, and economy-of-movement indices), they trained and compared five ML algorithms to classify trials into two (beginning vs. end) and three (beginning vs. middle vs. end) phases of training progression. A support vector machine achieved accuracies of above 84% for the two-class and 86% for the three-class problems, reinforcing the potential for AI-based models to map learning trajectories over time.

### Behavioral and interaction analysis

This functional domain focuses on decoding complex human behaviors and team dynamics from observations (often multimodal). As the previous function, it often builds on outputs from Data Processing and Integration (e.g., transcripts, diarized audio segments, trajectories, temporal markers). AI methods in this domain may include NLP applied to transcripts (e.g., identifying communication content categories, teamwork behaviors) and CV applied to video (e.g., recognizing gestures or actions). Such analysis allows the identification of subtle, complex, or longitudinal patterns in individual and team behavior, that are often imperceptible through manual or conventional analysis methods [22, 24, 26, 31].

One illustrative example is provided by Mayes et al. [22] who combined NLP and ML to analyze communication during simulated pediatric trauma handoffs. Communications from operating room to ICU transfers were recorded, transcribed, and analyzed using an NLP pipeline paired with a support vector machine classifier. The AI model was trained to detect specific content categories (e.g., patient status, anesthesia details, surgical data) and teamwork-related behaviors (e.g., equipment checks, logistical coordination, environmental context) from the transcript of each handoff. The resulting automated categorization of communication patterns closely matched expert human ratings, enabling the creation of behavioral “fingerprints” for each team.

Similarly, CV-based pattern recognition has shown promise in surgical simulation contexts. Atroshchenko et al. [24] applied DL algorithms to video recordings of robotic cardiac surgical simulations to classify specific gestures (suturing and dissection) and assess technical skills (mitral stitches and the atrial closure). The architecture combined a convolutional neural network, to extract spatial features from each frame, with a long short-term memory layer, to capture temporal dependencies across frames. This enabled the model to learn kinematic patterns characteristic of expert versus novice performance. The action-recognition component achieved high accuracy and predictive certainty despite a relatively limited dataset, demonstrating that expert and novice gesture patterns can be reliably distinguished from raw video. The skill-assessment network showed promising but less stable performance, indicating that larger training datasets are needed before such models can be used for routine evaluation.

### Automation and acceleration

This functional domain targets the research workflow itself, using AI to automate or semi-automate tasks that traditionally limit the scale and timeliness of simulation studies. Examples include automating transcription and structuring of debriefing discourse, supporting qualitative coding workflows, clustering open-ended responses, drafting structured summaries, or generating draft versions of scenario documentation and checklists for expert review and adaptation [20, 35–39, 41]. LLMs and other AI tools are increasingly reported across education and health research as “human-in-the-loop” systems where AI proposes first-pass codes or drafts that are then checked and refined by researchers [20, 38, 39, 41]. Empirical work in qualitative research suggests that, when carefully prompted and supervised, LLMs can achieve agreement with human coders in the moderate-to-substantial range while dramatically reducing analysis time [20, 38, 39, 41].

Similar approaches are beginning to appear in simulation research workflows. Hong et al. [42] piloted an LLM-supported debriefing assistant in pediatric resuscitation simulations: real-time transcripts from each scenario were fed to GPT-4o to generate structured debriefing prompts and summary notes, with facilitators rating the tool as acceptable and reporting reduced perceived workload. Generative AI has also been used to accelerate scenario and virtual patients’ role development. Barra et al. [43] described an agentic AI workflow for healthcare simulation scenario design that evolved from a simple ChatGPT prototype into a multi-agent pipeline capable of generating, refining and documenting scenarios aligned with local learning objectives, reducing preparation time by up to 80%.

## From concept to application: using the framework as a planning tool

The proposed framework is intended primarily for the planning phase of AI-amplified research, helping teams make explicit and intentional decisions about whether and how to incorporate AI into their studies. It is designed as a reflective planning aid rather than a rigid checklist. The following five steps illustrate how researchers might embed the framework into study planning.

### Begin with the research question and analytic task

A first step is to formulate the research questions clearly and translate them into specific analytic tasks. For example, a project may seek to: compare performance across training conditions, detect emergent patterns of team communication, or scale qualitative analysis of debriefing transcripts. Clarifying *what decisions the analysis is intended to inform* is particularly helpful, as it encourages researchers to consider AI as a tool rather than as an end in itself. At this stage, it may also be useful to ask whether the problem genuinely requires AI, or whether conventional methods would suffice, given the available data and resources.

### Map the question to functional domains and planned applications

Once the analytic task is articulated, the framework can be used to locate the work within one or more functional domains (Fig. 2). After identifying the relevant functional domain(s), it is useful to make explicit the intended application within that domain, that is, what AI is expected to do in practical terms. Explicitly documenting these links in protocols or ethics applications can support clearer communication with collaborators/reviewers and transparent justification of AI use.

### Specify data sources, representations, and integration needs

After identifying the functional domain, researchers can use the framework to plan *what data are needed and in what form*. For some studies, especially those using a single structured data source, integration may be simple, whereas for team-based or multisite projects, early consideration of integration requirements improves data quality and the feasibility of downstream analyses. For each identified functional domain, helpful questions include:

- ▪ Which data sources are essential?
- ▪ What intermediate representations will be required?
- ▪ Is a dedicated Data Processing and Integration layer required to transform raw traces into these representations, or can existing manual processes suffice?

### Deliberately design the human–AI division of labor

Following the principles of accountability, transparency and ethical use of AI, the framework can also be used to explicitly consider *who (or what) does what* in the analytic workflow. Making these boundaries explicit in the study protocol can also support ethical review and team discussions about roles and responsibilities. Within each functional domain, researchers can decide which tasks might be:

- ▪ *Automated* (e.g., first-pass transcription, diarization, or extraction of basic simulator metrics);
- ▪ *AI-assisted with human-in-the-loop* (e.g., AI-suggested qualitative codes, draft debriefing summaries, or preliminary risk scores that are checked and interpreted by researchers); and
  ▪ *Reserved for human expertise* (e.g., high-stakes progression decisions, final interpretation of complex interaction patterns).

### Plan evaluation and reporting from the outset

Finally, the framework can support planning for evaluation and reporting of AI components. For each functional domain where AI is used, researchers may consider in advance how performance and impact will be assessed:

- ▪ For *Data Processing and Integration*, evaluation may focus on synchronization accuracy, error rates in automatic labeling, or agreement between automated and manual annotations.
- ▪ For *Comparative and Predictive Analytics*, established guidance such as TRIPOD + AI [44] and related reporting tools can inform validation strategies and transparent reporting of model development, performance, and generalizability.
- ▪ For *Behavioral and Interaction Analysis*, evaluation may include triangulation with expert ratings, sensitivity analyses to different modeling choices, among other indicators.
- ▪ For *Automation and Acceleration*, evaluation may include both methodological indicators (e.g., time saved, agreement with human coders) and educational or organizational outcomes (e.g., increased sample size, feasibility of multi-site designs).

To support implementation, a preliminary version of an AI-Amplified Data-Driven Simulation Research Planning Template (AID^2^SimRes) reflecting these steps is provided in the supplementary material (see Additional file 1).

## Considerations on ethics, trust and accountability

Integrating AI into data-driven simulation-based research amplifies analytics but is not without challenges and concerns about privacy, fairness, validity, and accountability. Because simulation often involves rich traces of identifiable participants, and AI outputs may inform assessment, feedback, or influence educational and clinical decisions, these considerations should be addressed during the research planning phase rather than retrospectively [14–16, 29, 45]. The key considerations below are presented in relation to each functional domain, reflecting how distinct risks and safeguards emerge across different AI-enabled research activities.

### Data processing and integration

Both multimodal and single-modality datasets can contain highly identifiable information and therefore require robust governance. Video and audio recordings, in particular, may capture faces, voices, names, and contextual details that make full anonymization difficult. Studies such as METS, TeamVision, and others illustrate how the collected data can be information-rich, increasing the risk of identification [7, 21, 26]. This level of data collection demands clear consent procedures. These should explicitly describe AI-based processing, potential secondary use, and data-retention periods. Safeguards are also needed when commercial or cloud-based services are used for storage or transcription, in line with institutional privacy policies and international regulations such as HIPAA and the EU AI Act [45]. In practice, this may include removing direct identifiers from structured data, pseudonymizing participants via coded IDs, and applying privacy-enhancing techniques to audiovisual material (e.g., face blurring, voice distortion, or replacing raw media with derived representations such as, pose skeletons or speaker-labeled segments). In multicenter collaborations, federated learning approaches can further reduce privacy risks by allowing models to be trained locally on institutional data while sharing only model parameters or learned features with a central aggregator, thereby preserving data ownership and limiting the transfer of raw identifiable data [46]. Such measures align with broader work on trustworthy and responsible AI, which emphasizes that technical design choices, governance processes, and clear communication about data handling contribute to sustaining trust in AI-enabled systems [45].

### Comparative and predictive analytics and behavioral and interaction analysis

Within the analytic domains, concerns focus particularly on bias, generalizability, and interpretability. Machine learning models for expertise classification or learning-curve prediction have demonstrated promising accuracy, but often rely on relatively small, single-center datasets [32–34]. In many simulation contexts, predictive performance may therefore be modest, and model outputs should be interpreted cautiously. Moreover, predictions may be misinterpreted as deterministic labels rather than probabilistic indicators, raising concerns if they are used to make high-stakes decisions about learners’ progression. To mitigate such risks, it is recommended that rigorous internal and external validation (e.g., cross-validation, temporal or external test sets), transparent reporting of model development, and use of checklists such as the Machine Learning to Assess Surgical Expertise tool [47]. Bias may arise from speech- and vision-based analytics as these models may perform differently across accents, languages, skin tones, or professional groups, as suggested by work on automatic speech recognition based debriefing analysis and multimodal teamwork assessments [31, 48]. To reduce the risk of unfair impact on particular learner groups, simulation researchers are encouraged to report training and test populations, examine performance across relevant subgroups where possible, and avoid relying on unvalidated models for high-stakes decisions [44].

### Automation and acceleration

LLMs and generative AI introduce a different set of challenges. Recent simulation studies have used LLMs to support debriefing, qualitative coding, summary generation, or scenario design, reporting substantial time savings and improved scalability [20, 42, 43]. However, these systems can also produce plausible but incorrect codes, clinical details, or feedback and may embed cultural or institutional biases in generated scenarios or debrief prompts [16, 35]. A human-in-the-loop approach can help preserve integrity and maintain trust in the resulting analyses.

## Conclusions

This paper proposes a function-based framework to support the purposeful use of AI as a research amplifier in data-driven simulation-based research. By organizing AI applications according to their function in the research workflow, the framework offers a stable conceptual structure that remains useful despite rapid changes in specific algorithms, platforms, and terminology. Applied upstream, it can help researchers clarify the analytic task, identify the kinds of data representations required, and make explicit choices about whether AI meaningfully adds value beyond conventional approaches.

Future work should focus on empirical testing and refinement of the framework across diverse simulation contexts, including multi-site and interprofessional settings, and on the development of practical resources (e.g., reporting templates, example pipelines) to support adoption by researchers with varying levels of technical expertise. As AI becomes increasingly embedded within simulation-based research, the central challenge is not whether AI can be used, but how to apply it intentionally and responsibly, and ultimately use it to amplify simulation-based research.

## Supplementary Information

Supplementary Material 1.

## Data Availability

No datasets were generated or analysed during the current study.

## Abbreviations

AI: Artificial Intelligence
CV: Computer Vision
DL: Deep Learning
EU AI Act: European Union Artificial Intelligence Act
HIPAA: Health Insurance Portability and Accountability Act
LLMs: Large Language Models
ML: Machine Learning
NLP: Natural Language Processing
VR: Virtual Reality
