# Supplementary material for: A function-based framework for AI-amplified, data-driven healthcare simulation research
Source: Adv Simul (Lond). 2026 Feb 28;11:22. doi: 10.1186/s41077-026-00426-x (PMC13001207; doi:10.1186/s41077-026-00426-x)
Supplement: Supplementary file 1 — Supplementary Material 1. [file 41077_2026_426_MOESM1_ESM.docx]

**AI-Amplified Data-Driven Simulation-Based Research Planning Template (v1.0)**

**Last Updated by** (name, date)**:** Click here to add text

**PROJECT/STUDY OVERVIEW**

| **Title** | | **Acronym** (if applicable) |
| --- | --- | --- |
| Click here to add text | | Click here to add text |
| **Brief Description** | | |
| Click here to add text | | |
| **Research Team and Roles** (include name, institution, background, specific role(s)) | | |
| PI/Research Lead | Click to add text | |
| Researchers | Click to add text | |
| Technicians/Assistants/Fellows | Click to add text | |
| Others | Click to add text | |

**1. RESEARCH QUESTIONS AND ANALYTIC TASKS**

*Add as many* ***Research Questions*** *as needed by copying the table below.*

| **Research Question #1** | **Key analytic task(s)** |
| --- | --- |
| Click here to add text | Click here to add text |
| **Rationale for using AI** *(vs conventional approaches)****:*** | |
| Click here to add text | |

**2. MAP THE QUESTION TO FUNCTIONAL DOMAINS, APPLICATIONS AND AI CAPABILITIES**

*Copy the research questions and analytic tasks from Section 1.*

| **Research Question** | | **Key analytic task(s)** | |
| --- | --- | --- | --- |
| Click here to add text | | Click here to add text | |
| **Functional domain** | **Planned AI application** | |  |
| **Data Processing and Integration** | e.g.,  *Format standardization*  *Feature extraction*  *Synchronize data streams*  *Automated quality assurance* | | |
| **Comparative and Predictive Analytics** | e.g.,  *Compare performance*  *Model trajectories*  *Predict outcomes* | | |
| **Behavioral and Interaction Analysis** | e.g.,  *Decode patterns*  *Detect trends* | | |
| **Automation and Acceleration** | e.g.,  *Automate repetitive tasks*  *Multi-site data analysis*  *Support large-scale analysis* | | |

**3. SPECIFY DATA SOURCES, REPRESENTATIONS, AND INTEGRATION NEEDS**

| **Data source** | **Needed representation /feature** | **Requires**  **Data Processing and Integration?** | **Notes on integration**  *(e.g., synchronization, quality checks)* |
| --- | --- | --- | --- |
| e.g., *simulator logs,* | e.g., *Summary metrics; event sequence;* | □ Yes  □ No |  |
| e.g., *video* | e.g., *Pose / gesture traces;* | □ Yes  □ No |  |
| e.g., *audio* | e.g., *ASR transcript; speaker-labelled segments* | □ Yes  □ No |  |
|  |  |  |  |

**4. HUMAN–AI DIVISION OF LABOUR**

*Copy the* ***AI Applications*** *from Section 2.*

| **AI Applications** | **Performed by** | **Specify any interactions and safeguards** |
| --- | --- | --- |
| e.g., data capture & preprocessing | □ Automated by AI  □ AI-assisted (human-in-the-loop)  □ Human-only |  |
| e.g., feature extraction | □ Automated by AI  □ AI-assisted (human-in-the-loop)  □ Human-only |  |
| e.g., transcription | □ Automated by AI  □ AI-assisted (human-in-the-loop)  □ Human-only |  |
|  |  |  |

**5. EVALUATION AND REPORTING**

| **Functional domain** | **Planned Evaluation**  (Specify how performance of AI components (e.g., prompts, models) will be measured) | **Documentation and Reporting**  (Specify how AI components (e.g., prompts, models) will be documented; Indicate which AI guidelines/checklists will be followed) |
| --- | --- | --- |
| **Data Processing and Integration** |  |  |
| **Comparative and Predictive Analytics** |  |  |
| **Behavioral and Interaction Analysis** |  |  |
| **Automation and Acceleration** |  |  |
